# Versatile Design of NO‐Generating Proteolipid Nanovesicles for Alleviating Vascular Injury

**DOI:** 10.1002/advs.202401844

**Published:** 2024-06-17

**Authors:** Yueyue Yang, Xiangyun Zhang, Hongyu Yan, Rongping Zhao, Ruixin Zhang, Liuyang Zhu, Jingai Zhang, Adam C. Midgley, Ye Wan, Songdi Wang, Meng Qian, Qiang Zhao, Ding Ai, Ting Wang, Deling Kong, Xinglu Huang, Kai Wang

**Affiliations:** ^1^ Key Laboratory of Bioactive Materials for the Ministry of Education College of Life Sciences Nankai University Tianjin 300071 China; ^2^ School of Medicine Nankai University Tianjin 300071 China; ^3^ First Central Clinical College Tianjin Medical University Tianjin 300192 China; ^4^ Department of Physiology and Pathophysiology Tianjin Medical University Tianjin 300070 China; ^5^ Tianjin Key Laboratory of Urban Transport Emission Research College of Environmental Science and Engineering Nankai University Tianjin 300071 China

**Keywords:** cell‐mimicking, endothelialization, nanozymes, proteolipid nanovesicles, vascular injury

## Abstract

Vascular injury is central to the pathogenesis and progression of cardiovascular diseases, however, fostering alternative strategies to alleviate vascular injury remains a persisting challenge. Given the central role of cell‐derived nitric oxide (NO) in modulating the endogenous repair of vascular injury, NO‐generating proteolipid nanovesicles (PLV‐NO) are designed that recapitulate the cell‐mimicking functions for vascular repair and replacement. Specifically, the proteolipid nanovesicles (PLV) are versatilely fabricated using membrane proteins derived from different types of cells, followed by the incorporation of NO‐generating nanozymes capable of catalyzing endogenous donors to produce NO. Taking two vascular injury models, two types of PLV‐NO are tailored to meet the individual requirements of targeted diseases using platelet membrane proteins and endothelial membrane proteins, respectively. The platelet‐based PLV‐NO (pPLV‐NO) demonstrates its efficacy in targeted repair of a vascular endothelium injury model through systemic delivery. On the other hand, the endothelial cell (EC)‐based PLV‐NO (ePLV‐NO) exhibits suppression of thrombosis when modified onto a locally transplanted small‐diameter vascular graft (SDVG). The versatile design of PLV‐NO may enable a promising therapeutic option for various vascular injury‐evoked cardiovascular diseases.

## Graphical abstract

Developing new strategies to alleviate vascular injury remains a challenge. Herein, biomimetic NO‐generated proteolipid nanovesicles (PLV‐NO) are introduced that recapitulates cell‐mimicking functions for vascular repair and replacement. The versatile design of PLV‐NO, utilizing membrane proteins derived from different cell types such as platelet and endothelial cells, offers a promising therapeutic option for various cardiovascular diseases induced by vascular injury.

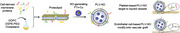

## 1 Introduction

Cardiovascular disease (CVD) remains a prominent global cause of mortality.^[^ ^1^ ^]^ The pathogenesis and progression of CVD are significantly influenced by vascular injury, representing trauma that affects the blood vessels responsible for transporting blood to specific organs or tissues.^[^ ^2^ ^]^ Vascular injury includes both endogenous chronic injury and traumatic injury. Endogenous injury is typically induced by major pathophysiological processes, such as endothelial dysfunction, atherosclerosis (AS), and subclinical inflammation,^[^ ^3^ ^]^ while traumatic injury can result from various surgical procedures or accidents.^[^ ^4^ ^]^ Different types of intervention strategies are applied based on the severity of the vascular injury. For example, in the case of vascular endothelial injury caused by surgical operations, such as arteriography and percutaneous coronary intervention (PCI), the repairment of the endothelial cell (EC) becomes a feasible treatment option, as the absence of endothelium can lead to various vascular complications, including thrombosis, inflammation, intimal hyperplasia (IH), and potentially arterial occlusion.^[^ ^5^ ^]^ On the other hand, in the instances of severe vascular injuries leading to a complete blockade owing either to the endogenous lesions or exogenous trauma, the implantation of vascular grafts becomes imperative. Vascular grafts, especially, small diameter vascular grafts (SDVGs), often face challenges, such as thrombosis and stenosis, in the absence of ECs on the lumen surface of grafts.^[^ ^6^ ^]^ Therefore, the development of various intervention strategies to alleviate vascular injury is essential, with a primary focus on promoting endothelialization in injured vessels.

Nitric oxide (NO), a central endogenous gas signaling molecule produced by various cells, plays a crucial role in modulating the functions of ECs, thereby facilitating the promotion of endothelialization in injured vessels.^[^ ^7^ ^]^ A myriad of NO‐generating stent coatings, including hydrogel containing organoselenium (SeCA),^[^ ^8^ ^]^ copper ions (Cu^2+^)‐chelated DOTA (1,4,7,10‐tetraazacyclododecane‐N,N′,N″,N″′‐tetraacetic acid)^[^ ^9^ ^]^ or dopamine‐copper (DA‐Cu) network^[^ ^10^ ^]^ have been developed to alleviate vascular injuries from angioplasty and stent deployment. These coatings catalytically decompose endogenous S‐nitrosothiols (R'SNO) to produce NO, thereby promoting anti‐thrombogenesis and rapid endothelium restoration. In addition, Zhao et al. leveraged nitrate‐functionalized prosthesis^[^ ^11^ ^]^ or galactosidase (an enzyme for NO prodrug conversion)^[^ ^12^ ^]^ which released NO to enhance the anti‐thrombogenicity and patency of vascular grafts. Consequently, developing approaches to mimic cellular NO production holds great promise for alleviating vascular injury. Recently, the bottom‐up construction of cell‐mimicking lipid vesicles capable of encapsulating functional molecules provides the possibility to realize these objectives.^[^ ^13^ ^]^ However, the construction of ideal cell‐mimicking vesicles that not only recapitulate the required functions of specific cells but also produce an appropriate amount of NO by catalyzing its endogenous donors remains challenging.

**Figure 1:**
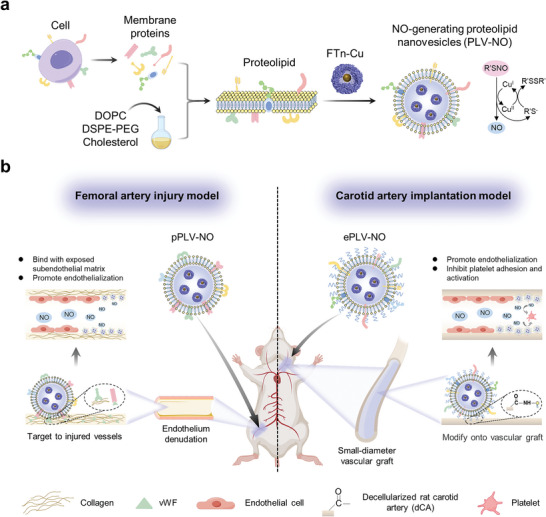
Design of PLV‐NO for vascular repair and replacement. a) Schematic illustration of the biomimetic construction of PLV‐NO. PLV‐NO were prepared by reconstituting membrane proteins from specific cells into lipid nanovesicles to form PLV, followed by the encapsulation of nanozymes (i.e., FTn‐Cu) capable of catalyzing endogenous donors to produce NO. b) Versatile design of PLV‐NO for two application scenarios of vascular injury. To target injured vessels via systemic delivery, pPLV‐NO was tailored by reconstituting platelet‐derived membrane proteins into lipid nanovesicles. The released NO from pPLV‐NO facilitated the repair of vascular injured sites following intravenous injection into a wire‐mediated rat femoral artery injury model. ePLV‐NO was tailored for inhibiting platelet aggregation by reconstituting EC‐derived membrane proteins into lipid nanovesicles. The ePLV‐NO was immobilized onto the lumen of dCA via an amidation reaction between the carboxylic (‒COOH) groups on dCA and the amino (‒NH_2_) groups on ePLV‐NO (i.e., dCA@ePLV‐NO). The NO released from ePLV‐NO allowed for the suppression of thrombosis and promotion of endothelialization following implantation into a rat carotid artery model.

In this study, we developed a NO‐generating proteolipid nanovesicle (PLV‐NO) technology that mimics the required functions of specific cells by reconstituting cell‐based proteolipid nanovesicles alongside packaging NO‐generating nanozymes. We assessed the salvage effect of PLV‐NO on the functions of ECs, including cell migration and neovascularization. On the basis of the benefits of PLV‐NO in personalized design, we tailored two types of PLV‐NO by reconstituting platelet‐ and EC‐derived membrane proteins into lipid nanovesicles. By harnessing the targeting ability of platelets toward injured vessels,^[^ ^14^ ^]^ we investigated the targeting capacity as well as repair effect of intravenously‐delivered platelet‐based PLV‐NO (pPLV‐NO) in a wire‐mediated rat femoral artery injury model. Based on the anti‐thrombotic function of ECs,^[^ ^15^ ^]^ we delineated the potential of EC‐based PLV‐NO (ePLV‐NO) in improving the success rate of SDVG post‐implantation. The promotion of endothelialization in injured vessels was highlighted to be assessed in these two examples of vascular injury applications (**Figure** **1**).

## 2 Results and Discussion

### 2.1 Reconstruction of NO‐Generating Proteolipid Nanovesicles (PLV‐NO)

**Figure 2:**
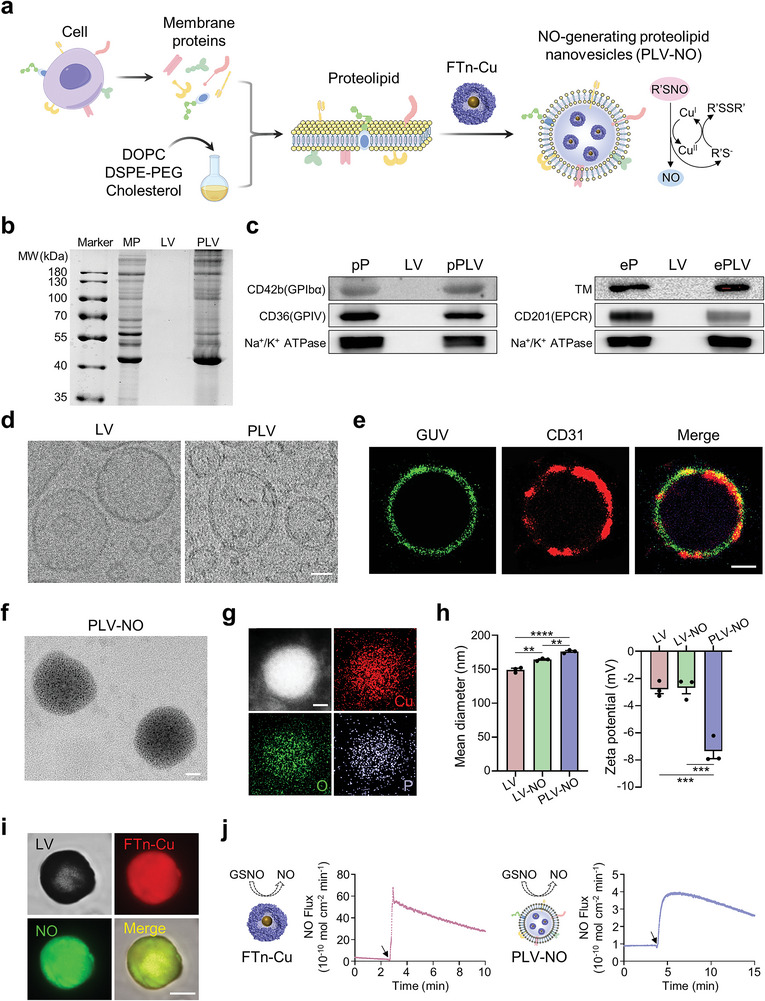
Preparation and characterization of PLV‐NO. a) Schematic illustration of the reconstruction of PLV‐NO by integration lipids, membrane proteins, and NO‐generating nanozymes (FTn‐Cu). b) Representative gel image of the protein compositions of the extracted membrane proteins (MP), LV, and PLV by SDS‐PAGE analysis. c) Western blot analysis of specific makers of CD42b/CD36 in pPLV and TM/CD201 in ePLV. d) Cryo‐electron microscopy images of LV and PLV. Scale bar = 50 nm. e) Confocal images of EC‐derived membrane proteins embedded into micro‐sized vesicles. Green, DiO‐labeled lipid layers; Red, PE‐labeled anti‐CD31 antibody. Scale bar = 5 µm. f) TEM image of PLV‐NO by direct observation without negative staining. Scale bar = 50 nm. g) Scanning TEM (STEM) image and corresponding elemental mapping images of PLV‐NO. Scale bar = 50 nm. h) Quantification analysis of mean diameters and zeta potentials of LV, LV‐NO, and PLV‐NO (n = 3 per group). i) Representative images of micro‐sized PLV‐NO particles via observation with confocal microscopy. Red, Cy5‐labeled FTn‐Cu; green, NO‐sensitive probe. Scale bar = 5 µm. j) Real‐time monitoring of NO release from 10 µm GSNO catalyzed by nanozymes and PLV‐NO using chemiluminescence NO analyzer. Arrow indicates the introduction of nanozymes or PLV‐NO. ^**^p < 0.01; ^***^p < 0.001; ^****^p < 0.0001. All data are presented as means ± SEM. One‐way ANOVA was used to compare between three groups.

Typically, the construction of PLV‐NO involved the reconstitution of synthetic phospholipids and membrane proteins to form proteolipid nanovesicles (PLV), followed by the encapsulation of NO‐generating nanozymes into PLV (**Figure** **2**a). For reconstitution of PLV, membrane proteins were initially extracted from specific cells based on the requirements and were subsequently embedded into lipid nanovesicles (LV) at a mass ratio of membrane proteins to synthetic phospholipids of 1:300 using thin‐film hydration and extrusion technology. The SDS‐PAGE analysis revealed similar protein compositions between the extracted membrane proteins and PLV (Figure 2b). Following reconstitution of the PLV with membrane protein from platelets (pP) and ECs (eP), western blot analysis confirmed the presence of pP and eP in the resulting PLV, as evidenced by the specific makers of CD36/CD42b and TM/CD201, respectively (Figure 2c). High‐resolution cryo‐electron microscopy (EM) images exhibited slight changes in lipid bilayers compared to LV, which was ascribed to the anchoring of membrane proteins (Figure 2d). These results confirmed the successful reconstitution of membrane proteins into the LV. To evaluate whether membrane proteins maintain their inherent orientation after anchoring into PLV, EC‐derived membrane proteins were embedded into giant unilamellar vesicles (GUV) using the same reconstitution method. A subsequent staining with PE‐labeled anti‐CD31 antibody and confocal imaging revealed the correct orientation of membrane proteins after reconstitution, as indicated by the distribution of CD31 molecules onto DiO‐labeled lipid layers (Figure 2e).

To further construct PLV‐NO, a NO‐generating nanozyme was incorporated into PLV using thin‐film hydration and extrusion technology. The nanozymes were prepared via in situ incorporation of Cu nanoclusters into the hollow cavity of genetically engineered human ferritin heavy chain (FTn), with a diameter of ≈12 nm (Figure S1a, Supporting Information). Transmission electron microscope (TEM) images displayed the hollow structure of FTn after negative staining and manifested Cu nanoclusters‐containing FTn (FTn‐Cu) (Figure S1b, Supporting Information). After loading into PLV, the NO‐generating nanozymes were directly observed under TEM (Figure 2f), and the elemental mapping of copper further confirmed the uniform distribution of FTn‐Cu in PLV (Figure 2g). Dynamic light scattering (DLS) analysis indicated an increase in the size of LV after the incorporation of nanozyme as well as membrane protein (Figure 2h). In contrast, changes in the zeta potential of LV were only observed upon anchoring membrane proteins. Additionally, the stability of PLV‐NO in different buffers was analyzed. There were insignificant changes in the particle size either in the PBS or 10% fetal bovine serum (FBS) solution overtime at 4 °C, thereby indicating that the PLV‐NO was stable at least for up to 7 days (Figure S2, Supporting Information). The catalytic ability of FTn‐Cu was evaluated by inducing NO production via the decomposition of endogenous NO donor S‐nitrosothiols (R'SNO). In this reaction, the NO is released by the R'SNO via Cu^I^‐mediated catalysis, which results in the production of Cu^II^. The generated Cu^II^ is further converted to Cu^I^ under reduction by L‐glutathione (GSH), allowing for the perpetual catalysis of the R'SNO.^[^ ^16^ ^]^ In the simultaneous presence of the S‐nitrosoglutathione (GSNO) and GSH, different concentrations of FTn‐Cu significantly induced NO production, as analyzed by Griess assays (Figure S1c, Supporting Information). To visualize NO production, Cy5‐labeled FTn‐Cu was encapsulated into microsized PLV. Confocal images manifested distinct NO production (green) as observed in the presence of NO probes and donors (Figure 2i). Additionally, the release kinetics of NO catalyzed by the nanozymes and PLV‐NO were compared by real‐time monitoring of NO release using a chemiluminescence NO analyzer. After the addition of the FTn‐Cu and PLV‐NO, the NO production was immediately observed and gradually decreased with the continuous consumption of GSNO (Figure 2j). Importantly, as compared to the highest NO flux of FTn‐Cu (ca. 68 × 10^−10^ mol min^−1^), PLV‐NO exhibited significantly lower NO flux (ca. 4 × 10^−10^ mol min^−1^), which is similar to the NO release of natural ECs.^[^ ^17^ ^]^

We next examined whether the NO released through the catalysis of PLV‐NO can affect ECs behaviors as well as vascular functions. First, a Transwell assay was conducted to evaluate the migration ability of HUVECs, which were cultured in the upper chamber while various treatments were introduced into the lower chamber along with the NO donors (10 µm GSH and 10 µm GSNO). As compared to the other treatments, PLV‐NO with NO donors resulted in a noticeable improvement in the migration capacity of HUVECs (Figure S3, Supporting Information). Subsequently, in vitro pro‐angiogenic potential was evaluated using a tube formation assay. HUVECs were cultured on a solidified Matrigel for up to 12 h and were stained with FITC‐phalloidin. The capillary‐like structures formed after PLV‐NO treatment supplemented with NO donors were 1.6‐fold higher than that of all other treatments, which was consistent with the results of the Transwell migration assay (Figure S4, Supporting Information). Furthermore, an arterial ring test was performed to validate the vasodilation function of PLV‐NO. Following potassium chloride (KCl) mediated vasoconstriction, the addition of PLV‐NO with a donor induced a rapid decrease in tension force. In comparison, no obvious change was observed with the addition of PLV‐NO alone. Typically, PLV‐NO accompanied by the NO donor reduced tension force of ≈1.00 g alongside a vessel relaxation of ≈32.67% (Figure S5, Supporting Information). These results suggested that the PLV‐NO can facilitate vasodilation by catalyzing the release of NO from NO donors.

### 2.2 Preparation of pPLV‐NO for Targeting to Injured Vessels

**Figure 3:**
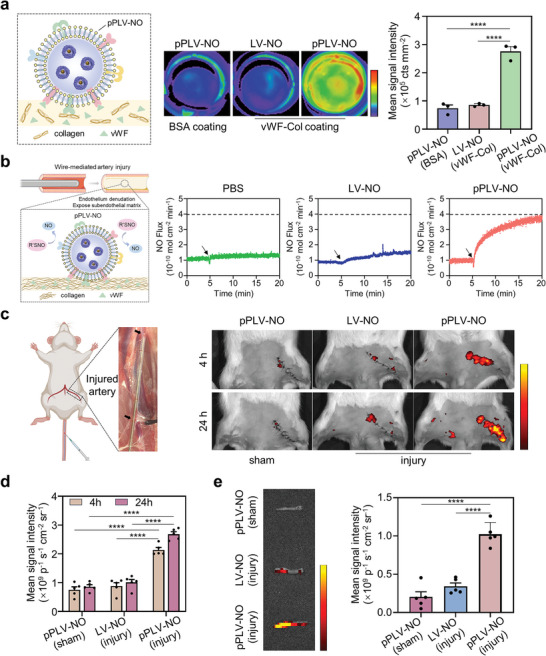
In vitro and in vivo targeting capacity of pPLV‐NO. a) Fluorescent imaging (middle) of vWF‐collagen or BSA coated surfaces after incubating with DiD‐labeled LV‐NO or pPLV‐NO. Quantitative analysis (right) was conducted by evaluating fluorescence intensities of the coated surfaces (n = 3 per group). b) Real‐time monitoring of NO release from GSNO catalyzed by different injured vessels incubated with PBS, LV‐NO, or pPLV‐NO, respectively. c) In vivo fluorescence images and d) quantification analysis of the accumulation of DiD‐labeled LV‐NO and pPLV‐NO at 4 and 24 h using IVIS Lumina imaging system (n = 5 per group). e) Ex vivo fluorescence imaging (left) and quantification analysis (right) of injured or sham arteries 24 h after administration (n = 5 per group). ^****^p < 0.0001. All data are presented as means ± SEM. One‐way ANOVA was used to compare between three groups. Multiple comparisons across two variables in figure d were performed using a two‐way ANOVA.

Cell membranes derived from the different types of cells offer multifunctional benefits in designing biomimetic nanomedicines.^[^ ^18^ ^]^ Notably, the coating of the nanomedicines with platelet membranes has been shown to significantly enhance their delivery to injured vessels, which is primarily attributed to the binding of their membrane proteins in the vascular injury sites.^[^ ^14^ ^]^ Consequently, to construct nanovesicles capable of targeting injured vessels through systemic delivery, we designed PLV‐NO by reconstituting platelet membrane proteins into NO‐generating LV, designated as pPLV‐NO. Upon vascular injury, subcutaneous matrix components, such as collagen and von Willebrand factor (vWF) are exposed, which leads to the recruitment of platelets. Various platelet surface molecules such as glycoprotein (GP)VI, GPIV, GPIb, GPIX, GPV, and GPIIb/IIIa play a crucial role in the process of platelet recruitment.^[^ ^14d^ ^]^ To assess the targeting efficiency of pPLV‐NO in vitro, glass slides were coated with vWF‐collagen to mimic vascular injury sites. After incubation on these coated glass slides, pPLV‐NO exhibited a robust binding ability, ≈3.2‐ and 3.7‐fold higher than that of the LV‐NO (with vWF‐collagen coating) and pPLV‐NO (with BSA coating), respectively (**Figure** **3**a). Confocal images further revealed that the occupied area by pPLV‐NO on vWF‐collagen coated surfaces was 4.6‐fold higher than that of LV‐NO, whereas the occupied area by pPLV‐NO on BSA‐coated surfaces was comparable to that of the LV‐NO on vWF‐collagen coated surfaces (Figure S6, Supporting Information).

We next sought to discern whether pPLV‐NO could specifically bind to injured vessels ex vivo, thereby leading to effective in situ catalysis of NO production. The ex vivo endothelium injury model was established by wire‐mediated scratching in the abdominal aorta isolated from rats. After incubation with or without nanovesicles, the released NO from the arteries was monitored in real‐time in the presence of donors using a chemiluminescent NO analyzer. As depicted in Figure 3b, upon the addition of donors, a burst NO release was observed in the injured arteries incubated with pPLV‐NO, reaching a plateau after 10 min. In contrast, injured arteries treated with LV‐NO only exhibited minute NO production.

Subsequently, the in vivo targeting of pPLV‐NO was evaluated in a wire‐mediated artery injury model. After intravenous administration of DiD‐labeled pPLV‐NO or LV‐NO, the injured arteries were imaged using an IVIS imaging system (Figure 3c). We observed an elevated fluorescence signal in the vascular injury sites after the injection of pPLV‐NO than that of the sham and LV‐NO‐treated groups. Image‐based quantification analysis revealed that the targeted pPLV‐NO in injured arteries at 24 h was 2.7‐ and 3.1‐fold higher than that of LV‐NO and the sham group, respectively (Figure 3d). Subsequently, major organs and injured arteries were isolated at 24 h for the analysis of in vivo biodistribution. We found that the administered particles were primarily distributed in the liver and spleen. Importantly, the signal intensity of pPLV‐NO in injured arteries was approximately threefold stronger than that of LV‐NO, which was close to the mean intensity of the liver and spleen (**Figure** **3**e; Figure S7a, Supporting Information). These results underscore the importance of anchoring platelet membrane protein into LV for targeting the injured blood vessels. In addition, the blood circulation profile of LV‐NO and pPLV‐NO was investigated via time‐dependent evaluation of the fluorescence intensity of blood after intravenous injection of DiD‐labeled nanovesicles into healthy rats.^[^ ^19^ ^]^ The blood circulation half‐life of pPLV‐NO was significantly longer than that of LV‐NO, indicating that the modification of platelet membrane proteins extends the blood circulation time of pPLV‐NO to some extent (Figure S7b, Supporting Information).

### 2.3 Repair of Injured Arteries by pPLV‐NO

**Figure 4:**
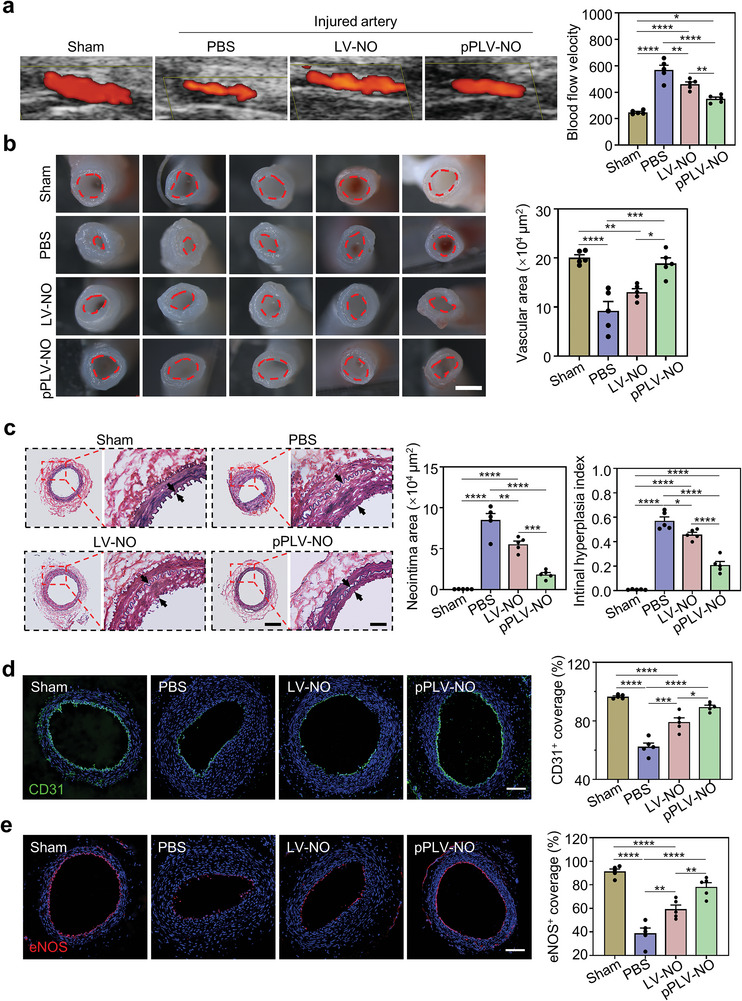
Repair of injured arteries with pPLV‐NO. a) The patency and blood flow of injured arteries in sham, PBS, LV‐NO, and pPLV‐NO groups were observed by color Doppler ultrasound following systemic administration (n = 5 per group). b) Stereomicroscope images of injured arteries in different treatment groups after 21 days. Vascular lumen area was quantified. Scale bar = 500 µm. c) VVG staining of cross sections of injured arteries in different groups, the neointima area and intimal hyperplasia index were calculated based on the VVG staining to reflect the histopathological characteristics of injured arteries. Three sections per sample and five samples per group were included for quantitative analysis. Scale bars are 200 µm (left) and 50 µm (right). d) Re‐endothelialization of injured arteries in different treatment groups were analyzed by anti‐CD31 immunofluorescence staining. Quantification of CD31^+^ cells coverage rate was performed. Scale bar = 100 µm. e) Re‐functionalized endothelialization of injured arteries was analyzed by immunofluorescence staining with anti‐eNOS. Three sections per sample and five samples per group were included for quantitative analysis of CD31^+^ and eNOS^+^ cells coverage rate. Scale bar = 100 µm. ^*^p < 0.05; ^**^p < 0.01; ^***^p < 0.001; ^****^p < 0.0001. All data are presented as means ± SEM. One‐way ANOVA was used to compare between four groups.

Next, we sought to delineate whether the repair of injured arteries by targeted pPLV‐NO was attributable to their NO production capacity. The rats subjected to wire‐mediated femoral artery injury were administrated with PBS, LV‐NO, or pPLV‐NO through tail vein injection. At day 21, color Doppler ultrasound imaging of injured arteries manifested varying extents of lumen stenosis in different groups (**Figure** **4**a). Quantification analysis of blood flow velocity, an index of lumen stenosis, illustrated that the degree of vascular stenosis of PBS group was 2.3‐fold higher than that of the sham group. The LV‐NO and pPLV‐NO treatments significantly alleviated stenosis, with a reduction for up to 80.87% and 61.52% of PBS group, respectively. After the isolation of injured arteries at day 21, stereo microscopy images and image‐based quantification analysis further confirmed the positive role of pPLV‐NO in alleviating vascular stenosis (Figure 4b). Histological analysis for elastin staining was performed to assess internal/external elastic lamina and neointimal hyperplasia. Extensive neointima hyperplasia was observed in PBS and LV‐NO treated group, whereas pPLV‐NO treatment significantly reduced this effect (Figure 4c). Quantification analysis demonstrated that the neointima area of pPLV‐NO treatment was reduced to 0.2‐ and 0.3‐fold of PBS treatment and LV‐NO treatment, respectively. The intimal hyperplasia index of pPLV‐NO‐treated group was 0.21 ± 0.03, which was less than 0.57 ± 0.03 of PBS treated group and 0.46 ± 0.02 of LV‐NO treated groups.

We next evaluated endothelium denudation and damage, which are the most direct evidence of vascular injury. Immunofluorescence staining for CD31 revealed that the pPLV‐NO‐treated arterial lumen was almost completely covered by the CD31^+^ ECs (Figure 4d). Image‐based quantification analysis revealed that the coverage rates of the CD31^+^ ECs in PBS and LV‐NO groups were 62.35% and 79.23%, respectively. In contrast, the pPLV‐NO group had reached ≈90% coverage by the CD31^+^ ECs, like the natural healthy arterial endothelium. Moreover, endothelial NO synthase‐positive (eNOS^+^) cells reflect the recovery of functional endothelium. As shown in Figure 4e, the treatment with pPLV‐NO promoted the recovery of eNOS^+^ ECs to 78.19% ± 3.71% of coverage rate, which was significantly higher than that of the PBS (38.87% ± 4.45%) and LV‐NO treated group (59.30% ± 3.55%). The morphology and phenotype of vascular smooth muscle cells (VSMCs) were evaluated by immunofluorescent staining of alpha smooth muscle actin (α‐SMA) and smooth muscle myosin heavy chain I (MYH) (Figure S8, Supporting Information). The VSMCs exhibited a circumferential alignment in healthy arteries (sham group), whereas a disordered arrangement in PBS‐treated injured arteries. The pPLV‐NO treatment significantly recovered the circumferential alignment of the VSMCs while LV‐NO lacked this recovery in the cell alignment (Figure S8a, Supporting Information). The cell nuclei alignment rate and nuclear shape index of 𝛼‐SMA^+^ VSMCs in pPLV‐NO group was approximately twofold higher than those of PBS group and ≈1.5‐fold higher than those of LV‐NO group (Figure S8b,c, Supporting Information). As compared to PBS and LV‐NO groups, pPLV‐NO significantly reduced the thickness of 𝛼‐SMA^+^ SMCs layer (Figure S8d, Supporting Information). Correspondingly, the recovery of contractile MYH^+^ VSMC showed a similar trend as it was observed in the circumferential alignment of VSMC (Figure S8e, Supporting Information). Additionally, no significant systemic toxicities were observed after the systemic administration of nanovesicles, as evidenced by hemolysis and H&E staining analysis (Figure S9, Supporting Information).

### 2.4 Preparation of ePLV‐NO for dCA Modification

**Figure 5:**
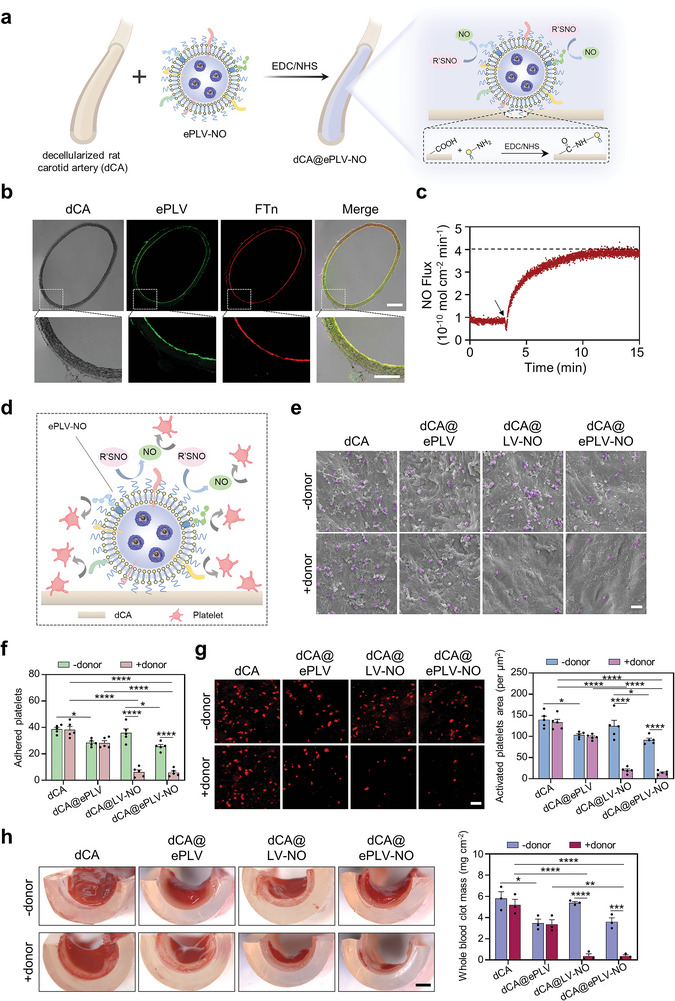
Preparation and characterization of ePLV‐NO modified dCA. a) Schematic illustration of the coating of dCA with ePLV‐NO via an amidation reaction between the carboxylic (‒COOH) groups on dCA and the amino (‒NH_2_) groups on ePLV‐NO (i.e., dCA@ePLV‐NO). b) Confocal images of the cross‐sections of dCA@ePLV‐NO. The ePLV and nanozymes were labeled with DiI and Cy5, respectively. Scale bars are 200 µm (top) and 100 µm (bottom). c) Real‐time monitoring of the release of NO from GSNO catalyzed by dCA@ePLV‐NO evaluated using chemiluminescence NO analyzer. d) Diagram illustrating the released NO from the surface of dCA@ePLV‐NO to inhibit platelet adhesion. e) SEM images of the adhesion of platelets to the lumen of differentially modified dCAs after incubation at 37 °C for 1 h in the presence or absence of donors. Scale bar = 5 µm. f) Quantitative analysis based on the number of adherent platelets on vascular lumen. Five samples per group and five randomly selected fields per sample were used for statistical analysis. g) Confocal images and quantitative analysis of the CD62P^+^ area after immunostaining. Five samples per group and five randomly selected fields per sample were evaluated for statistical analysis. Scale bar = 5 µm. h) Stereomicroscopic images of the clot of recalcified whole blood on the lumen of dCAs with different modifications. Quantitative results were obtained by dividing the added weight by the lumen area (n = 3). Scale bar = 1 mm. ^*^p < 0.05; ^**^p < 0.01; ^***^p < 0.001; ^****^p < 0.0001. All data are presented as means ± SEM. Multiple comparisons across two variables were performed using a two‐way ANOVA.

The implantation of SDVGs is a choice for severe arterial injuries, however, it is prone to the risk of acute thrombosis post‐implantation. Native endothelium plays an important role in maintaining vascular patency, which is mainly attributed to its unique membrane composition and secreted functional agents. The EC‐derived membrane protein as well as NO have been shown to promote anti‐thrombotic via the suppression of platelet aggregation.^[^ ^9^, ^12^ ^]^ As such, we designed a functionalized SDVG coated with NO‐generating EC‐based PLV (i.e., ePLV‐NO). In a typical procedure, EC‐derived membrane protein was initially reconstituted into LV, followed by their loading with NO‐generating nanozymes. Then, the ePLV‐NO was immobilized onto the lumen of decellularized rat carotid artery (dCA) via an amidation reaction between the carboxylic (‒COOH) groups on dCA and the amino (‒NH_2_) groups on ePLV‐NO (i.e., dCA@ePLV‐NO) (**Figure** **5**a). To visualize the modification effect, DiI and Cy5 were used to label ePLV and nanozymes, respectively. Confocal images of the cross sections of dCA@ePLV‐NO illustrated a uniform distribution of both ePLV and nanozymes in the inner layer of dCA (Figure 5b), indicating that the nano‐sized coating enabled an effective coverage of the lumen of dCA. We next deciphered whether dCA@ePLV‐NO could facilitate NO production in the presence of NO donor using a chemiluminescence NO analyzer. As shown in Figure 5c, dCA@ePLV‐NO steadily catalyzed the release of NO in the presence of 10 µM GSNO and 10 µM GSH. These results indicated that the modification of ePLV‐NO onto dCA can facilitate the functionalization of the grafts by mimicking the natural endothelium of vessels.

Considering platelet adhesion and activation on implanted grafts as the upstream essential steps for inducing thrombosis formation, we evaluated if ePLV‐NO can effectively inhibit platelet adhesion and activation in grafts. The adhesion ability of platelets to the lumen of the modified dCA was initially assessed (Figure 5d). Scanning electron microscopy (SEM) images (Figure 5e) and quantification analysis (Figure 5f) demonstrated that the modification of dCA with nanovesicles incorporating endothelial membrane protein (i.e., dCA@ePLV) inhibited platelet adhesion for up to some extent than that of the unmodified dCA. Moreover, increased production of NO significantly decreased the number of adherent platelets on dCA@LV‐NO and dCA@ePLV‐NO. Notably, there was an insignificant difference between the dCA@LV‐NO and dCA@ePLV‐NO groups in terms of platelet adhesion, which suggested the potency of the produced NO overshadowed the role of endothelial membrane proteins in reducing the aggregation of platelets. Subsequently, activated platelets on the dCA were assessed by immunofluorescence staining for CD62P. Confocal images alongside an image‐based quantification assay revealed the crucial role of endothelial membrane proteins and NO‐generating nanozymes in inhibiting the activation of platelets (Figure 5g), which was also in agreement with the results of platelet adhesion. Furthermore, the anti‐clot formation ability of modified dCAs was investigated using freshly prepared recalcified whole blood through a CaCl_2_‐evoked clotting approach. We found that dCA@ePLV, dCA@LV‐NO, and dCA@ePLV‐NO resulted in less blood clot formation than dCA alone, as evidenced by stereomicroscopic observation and clotting mass (Figure 5h). Importantly, these results indicate that endothelial membrane proteins and NO‐generating nanozymes exhibit a synergistic effect on anti‐thrombosis functions of dCA, and thus the dCA@ePLV‐NO was chosen as an optimal graft for further evaluation.

### 2.5 In Vivo Implantation of ePLV‐NO Modified dCA

**Figure 6:**
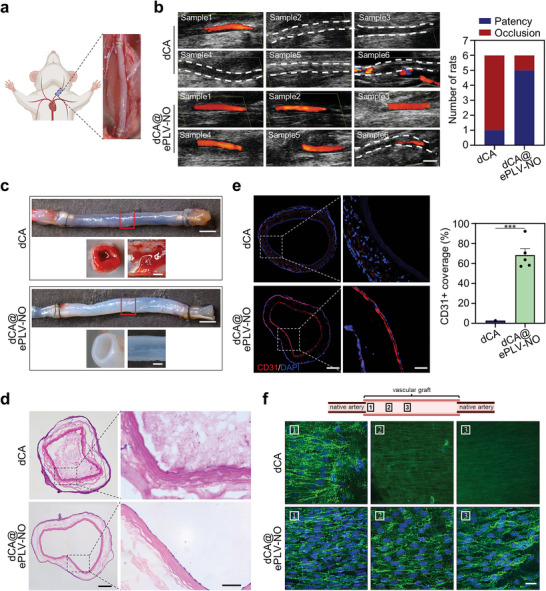
In vivo assessment of patency and endothelialization in rat carotid artery implantation models. a) Implantation of dCAs with or without the modification of ePLV‐NO into rat carotid artery using a “cuff” technique. b) Color ultrasound Doppler images depicting vascular patency of dCAs at 14 days post‐implantation (n = 6 per group). Scale bar = 2 mm. c) Representative stereomicroscopy images of the side view, cross‐section, and lumen of explanted grafts at 14 days. Scale bars are 2 mm (top) and 500 µm (bottom). d) Representative H&E staining images of the cross‐sections of midportion of explanted grafts. Scale bars are 200 µm (left) and 50 µm (right). e) Representative images and quantification analysis of endothelialization in explanted grafts following immunostaining for CD31. Three sections per sample, one patent sample in the dCA group and five patent samples in the dCA@ePLV‐NO group were included for statistical analysis. Scale bars are 200 µm (left) and 50 µm (right). ^***^p < 0.001. All data are presented as means ± SEM. T‐test was used to compare between two groups. f) Representative images of the en‐face staining for CD31 at three different sites (anastomotic, quarter, and midportion) of the internal lumen of the graft. Scale bar = 20 µm.

We next investigated if the modification of ePLV‐NO onto dCA (i.e., dCA@ePLV‐NO) can suppress thrombus formation in vivo. The dCA with or without ePLV‐NO modification were transplanted into rat carotid artery without an administration of anticoagulant (**Figure** **6**a). At 2 weeks post‐implantation, color ultrasound Doppler imaging revealed that five out of six transplanted dCA@ePLV‐NO grafts remained patent, showing no signs of aneurysm or stenosis (Figure 6b). In contrast, only one out of the six transplanted dCA grafts was patent, while the blood flow in the other implanted grafts was completely blocked. Subsequently, the grafts were harvested, and stereomicroscopy images demonstrated that the lumen surface of dCA@ePLV‐NO was clean and smooth, whereas significant thrombosis formation was observed in the unmodified dCA (Figure 6c). H&E staining of transverse sections indicated that the thrombus formation was the main cause of the occlusion in dCA. A confluent monolayer of cells was observed on the lumen of dCA@ePLV‐NO (Figure 6d), which were confirmed to be CD31‐positive ECs through immunofluorescence staining (Figure 6e). Quantitative analysis of graft sections showed that the modification of dCA with ePLV‐NO significantly improved the coverage rate of CD31‐positive ECs compared to unmodified grafts. To maintain good patency of vascular grafts, timely endothelialization of the lumen surface is pivotal to prevent thrombosis and IH. The endothelialization of the grafts was further examined at the anastomotic (position 1), quarter (position 2), and midportion sites (position 3) by immunofluorescent staining (Figure 6f) and SEM (Figure S10, Supporting Information). Representative confocal images revealed that the luminal surfaces at all three sites of the dCA@ePLV‐NO were fully covered by a confluent monolayer of CD31‐positive ECs exhibiting a cobblestone‐like morphology and elongation along the direction of blood flow.^[^ ^20^ ^]^ In contrast, for the dCA group with only one patency, ECs coverage was observed only at the anastomotic sites, while other sites were free of ECs. The results of ECs coverage were further confirmed by SEM observation (Figure S10, Supporting Information). These results indicated that ePLV‐NO modification could effectively improve anticoagulant properties as well as accelerate endothelialization of SDVGs.

## 3 Conclusion

In this study, we biomimetically constructed NO‐generating proteolipid nanovesicles by bottom‐up reconstituting cell‐derived membrane proteins into lipid nanovesicles for the encapsulation of protein‐based nanozymes. The reconstitution of proteolipid nanovesicles offers at least two aspects of benefits: i) exclusion of the complex cell components, particularly those with membrane lipid/nucleus‐relevant functions that may cause undesirable side effects; ii) flexible design of different cell‐derived proteolipid nanovesicles according to the requirement of the diseases. Using platelet membrane proteins and endothelial membrane proteins, the reconstituted proteolipid nanovesicles were applied for the systemic targeting of vascular endothelium injury and the modification of locally implanted SDVGs to reduce platelet adhesion/activation, respectively, which represent two clinically relevant examples of vascular injury. Thus, the use of membrane proteins derived from different types of cells holds promise for tailoring diverse regenerative treatments. However, at least two challenges associated with the preparation method of proteolipid nanovesicles need to be addressed before the bench‐to‐bedside translation, including the potential destruction of the inherent orientation of membrane proteins^[^ ^21^ ^]^ and large‐scale production under good manufacturing practice (GMP) standards.^[^ ^22^ ^]^ Additionally, the integration of NO‐generating nanozymes into proteolipid nanovesicles facilitates the migration and revascularization of ECs, thereby promoting the repair of injured vessels and inhibiting thrombus formation. Notably, the ultrasmall size and low nonspecific binding with biological molecules of these protein‐based nanozymes make them suitable for packing into proteolipid nanovesicles. Taken together, the ingenious design of nanozyme‐based proteolipid nanovesicles undoubtedly offers biomimetic alternatives and a new perspective for the treatment of vascular injury diseases.

## 4 Experimental Section

### Preparation and Characterization of FTn‐Cu

Recombinant human FTn was initially isolated from E.coli following the previous report.^[^ ^23^ ^]^ For the preparation of FTn‐Cu, the FTn was dissolved in PBS (2 mg mL^−1^) and the pH was adjusted to 8.5 using a 50 mm sodium hydroxide (NaOH) solution. The copper chloride (CuCl_2_) was then dissolved in deionized water (50 mg mL^−1^) and added to the FTn solution, resulting in a theoretical loading ratio of 1000 Cu/FTn. After stirring at 60 °C for 30 min, the solution was purified using a PD‐10 desalting column (GE Healthcare) to remove the unloaded copper ions (Cu^2+^). Sodium borohydride (NaBH_4_) was gently added to an ice bath for 2 h to reduce the Cu^2+^ loaded in the FTn to Cu^1+^. The concentration of FTn‐metal was determined using standard FTn samples as a reference by SDS‐PAGE gel electrophoresis analysis. The morphology of FTn or FTn‐Cu was observed by TEM (H7600, Hitachi), with or without the negative staining of the specimen with 1% uranyl acetate, respectively. NO generation from various concentrations of FTn‐Cu was evaluated at pH 7.4 in the presence of GSH (100 µm) and R'SNO (100 µm). The concentration of the released NO was subsequently assessed by a Griess Assay Kit.

### Preparation and Characterization of pPLV and ePLV

The membrane proteins of ECs and platelets were first extracted by Cell Membrane Protein Extraction Kit according to manufacturer instructions (Beyotime Biotechnology, China). Briefly, the cells were collected (1000 rpm, 3 min) and washed with PBS, then suspended in membrane protein extraction reagent A for a 30‐time homogenization in an ice bath. The supernatant was collected under centrifugation at 700 g (4 °C, 10 min) and the fragments of the cell membrane were precipitated after centrifugation at 14 000 g for 30 min. The pellets were suspended in membrane protein extraction reagent B and then the mixture was vortexed for 5 s and cooled in an ice bath for 10 min. Finally, the supernatant was collected under centrifugation at 14 000 g for 5 min and stored at −80 °C. For the reconstitution of membrane protein into lipid nanovesicles, a mixture of 1,2‐dioleoyl‐sn‐glycero‐3‐phosphocholine (DOPC), 1,2‐(DSPE‐PEG2000‐NH_2_), and cholesterol (Avanti Polar Lipids) were dissolved in chloroform, and the solvent was evaporated using a rotary evaporator to form a film. Afterward, the dried lipid film was hydrated with a PBS dispersion of membrane proteins (protein‐to‐lipid ratio of 1:300) or PBS to assemble PLV or LV, respectively, at 37 °C. This was followed by extrusion through 400, 200, and 100 nm polycarbonate filters for 20 cycles. The protein composition of ePLV or pPLV was characterized through SDS‐PAGE analysis. Moreover, the presence of marker proteins of pPLV (CD42b, CD36, and Na^+^/K^+^ ATPase) or ePLV (TM, CD201, and Na^+^/K^+^ ATPase) was confirmed using western blot analysis. For Cryo‐TEM analysis, LV and PLV were plunge‐frozen on holey film grids as previously reported.^[^ ^13^ ^]^ To further verify the correct orientation of membrane proteins on the vesicles, microsized PLV was prepared by reconstitution of EC‐derived membrane proteins into lipid vesicles. The resulting vesicles were labeled with DiO and the EC membrane protein was stained with CD31‐PE, followed by observation using confocal microscopy.

### Preparation and Characterization of PLV‐NO

To obtain FTn‐Cu loaded LV or PLV, the lipid film was fully hydrated with a 1 mL solution of FTn‐Cu (concentration, 2 mg mL^−1^) for 1 h at 37 °C. After removing the free FTn‐Cu through gradient centrifugation, the obtained pellet was resuspended with PBS. The resulting solution was extruded through 400, 200, and 100 nm polycarbonate filters for subsequent use. The presence of FTn‐Cu in the PLV was observed by TEM without negative staining with 1% uranyl acetate in the specimen. The elemental distribution of PLV‐NO was studied using a field emission TEM (FE‐TEM, Talos F200X G2, FEI).^[^ ^24^ ^]^ Particle sizes and zeta potentials were measured by dynamic light scattering (DLS) using a zeta‐sizer (Mastersizer 2000, Malvern). The stability of PLV‐NO was analyzed by incubation in PBS and 10% FBS at 4 °C and particle sizes were measured at the indicated time points. The FTn‐Cu‐mediated NO generation in the nanovesicles was monitored in the presence of NO working solution (100 µm GSH and 100 µm R'SNO) and DAF‐2 probes under fluorescence microscopy.

### Real‐Time Release of NO

The real‐time catalytic release of NO from specimens was monitored using a chemiluminescence NO analyzer (NOA) (Seivers 280i, Boulder, CO). Briefly, a 5 mL testing PBS solution consisting of 10 µm GSNO and 10 µM GSH, was initially added into the reaction chamber. Following a 5 min baseline calibration of NO levels, either the nanovesicles in PBS (0.5 mL) were injected into the reaction chamber or the arteries were immersed in the reaction chamber. N_2_ gas, with controllable airflow size, delivered NO to the NO analyzer. The quantity of generated NO was calculated based on the calibration curves of the NOA.

### Transwell Migration Assay

In the HUVEC Transwell migration assay, HUVECs (1 × 10^4^) were cultured in the upper chamber of the transwell inserts (pore size, 8 µm) and were placed onto a 24‐well plate. The collected medium containing PLV, PLV‐FTn, PLV‐NO (equivalent to 1 µg mL^−1^ of FTn‐Cu) with or without the NO donor (10 µm GSH and 10 µm GSNO) was directly added to the lower chamber. The cells were allowed to migrate for 12 h at 37 °C with 5% CO_2_. Thereafter, non‐migrated cells on the upper side of the membrane were removed. The migrated cells were fixed with 4% paraformaldehyde (PFA) for 15 min, followed by staining with 0.1% crystal violet for 30 min. The stained cells were observed and counted using microscopy.

### Tube Formation Assay

The in vitro pro‐angiogenic potential of the PLV‐NO was evaluated through a tube formation assay. Basement membrane matrix (Matrigel, BD Biosciences) was added to each well of a 48‐well cell culture plate and allowed to solidify through incubation at 37 °C for 30 min. Next, HUVECs (1 × 10^4^) were seeded on the solidified Matrigel. To discern the pro‐angiogenesis capacity of PLV‐NO, various treatments were introduced to the HUVECs, including PLV, PLV‐FTn, PLV‐NO (equivalent to 1 µg mL^−1^ of FTn‐Cu) with or without the donor (10 µm GSH and 10 µm GSNO) respectively. The medium without any nanovesicle served as a control. After co‐culturing for 12 h, the HUVEC‐lined vessels were washed with PBS, fixed with 4% PFA, and stained with FITC‐phalloidin. The capillary‐like structures were observed using confocal microscopy and quantified in randomly chosen fields using ImageJ software.

### In Vitro NO‐Mediated Vascular Vasodilation

The NO‐mediated vascular vasodilation was examined based on the previous method.^[^ ^23^, ^25^ ^]^ Briefly, the thoracic aorta was isolated from anesthetized SD rats and cut into rings (length, 4 mm) after the mechanical removal of the extraneous fat as well as connective tissues. The rings were then hung on the hooks connected to a force transducer in a bath containing 20 mL Krebs–Henseleit solution (118 mm NaCl, 4.7 mm KCl, 2.4 mm CaCl_2_, 1.2 mm KH_2_PO_4_, 2.4 mm MgSO_4_, 25 mm NaHCO_3_, 11.1 mm glucose, pH 7.4) at 37 °C under physiological O_2_ conditions (95% O_2_/5% CO_2_). Isometric forces were recorded by a force transducer connected to a PowerLab/870 Eightchannel 100 kHz A/D converter (AD Instruments, Sydney, Australia). After equilibration under a resting tension of 2.00 g for 1 h, KCl solution (60 mm) was added to induce the contraction of the rings. Once reached at an equilibrium tension, PLV‐NO (equivalent to FTn‐Cu at 1 µg mL^−1^) with or without the donor (100 µm GSNO and 100 µm GSH) were added into the bath solution. The tension force was recorded by the force transducer. The variations of tension force (Δ Tension) and the percentage of relaxation (Relaxation %) were defined as follows:

(1) ΔTensionforce=Fa−Fb

(2) $$\mathrm{Relaxation}\;\%=\left(\mathrm{Fa}-\mathrm{Fb}\right)/\mathrm{Fa}\times 100\%$$

where Fa and Fb represent the tension force before and after the addition of PLV‐NO, respectively.

### In Vitro Targeted Binding of pPLV‐NO to vWF‐Collagen

To replicate an injury‐site‐relevant subendothelial matrix surface presenting vWF and collagen, glass slides (n = 3) placed in a 48‐well polystyrene plate were coated with a solution of collagen (40 µg mL^−1^) and vWF (10 µg mL^−1^) in 20 µm acetic acid or 1% (w/v) BSA (negative control) overnight at 4 °C. Subsequently, the slides were washed with PBS to remove any excess coating solution. Then, 500 µL of DiD‐labeled LV‐NO or pPLV‐NO was added to each well and incubated at 37 °C for 1 h, followed by washing with PBS three times to remove any non‐adherent nanovesicles. The signal was collected using the in vivo Imaging System IVIS Luminar (Berthold Technologies, NightOWLIILB983), and the glass slides were observed with a confocal laser scanning microscope (Leica, TCS SP8) to assess the adhesion of LV‐NO or pPLV‐NO to the vWF‐collagen or BSA. Three images per sample and three samples per group were used to quantify the adherent nanovesicles.

### Ex Vivo Binding of pPLV‐NO to Injured Artery and In Situ NO Production

The abdominal aortas were harvested from rats, and the lumen was subjected to three times of injuries using a guide wire. Subsequently, the specimens were incubated with pPLV‐NO or LV‐NO. The control group received PBS. After 30 min incubation at 37 °C, the samples were washed three times with PBS to remove non‐adherent nanovesicles. The real‐time release of NO from arteries, in the presence of 10 µm GSNO and 10 µm GSH, was monitored using a chemiluminescence NOA (Seivers 280i, Boulder, CO) as described in the Experimental Section “Real‐time Release of NO”.

### Hemolysis Rate Analysis

To assess the biosafety of LV‐NO and pPLV‐NO, the hemolysis rate was measured. Fresh blood from healthy rat hearts was mixed with sodium citrate solution at a ratio of 9:1 and then added to saline at a volume ratio of 4:5 to prepare diluted anticoagulant blood. FTn‐Cu, LV‐NO, and pPLV‐NO, with or without NO donor (10 µm GSH and 10 µm GSNO), were added to 1 mL saline. Only saline was served as a negative control group, while distilled water was used as a positive control group. After incubation in a water bath at 37 °C for 30 min, 0.02 mL of diluted anticoagulant blood was added to all groups, mixed, and further incubated in a water bath at 37 °C for 60 min. The samples were then centrifuged at 800 g for 5 min, and the supernatant was collected to detect the absorbance at 545 nm. The hemolysis rate was calculated using the formula:

(3) $$\left(A-B\right)/\left(C-B\right)\times 100\%$$

where A is the absorbance of the sample, B is the absorbance of the negative control group, and C is the absorbance of the positive control group.

### In Vivo Assessment in Wire‐Mediated Rat Femoral Artery Injury Model

Sprague Dawley (SD) rats (male, 250–280 g, 8 weeks) were purchased from the Laboratory Animal Centre of the Academy of Military Medical Sciences (Beijing, China). All the animal experiments were approved by the Animal Experiments Ethical Committee of Nankai University (2022‐SYDWLL‐000189). Male SD rats were subjected to wire‐mediated vascular injury under anesthesia induced by intraperitoneal injection of pentobarbital sodium (30 mg kg^−1^ body mass), following a previously described protocol.^[^ ^26^ ^]^ Briefly, after tail vein injection of the anticoagulant heparin (100 units kg^−1^), femoral arteries were exposed through a longitudinal groin incision and monitored using a surgical microscope. To interrupt blood flow, a single knot was tied with 5‐0 silk thread around the femoral artery proximal to the inguinal ligament, and another knot was tied with 7‐0 silk thread around the femoral artery distal to the deep femoral branch. An incision hole was created distal to the deep femoral branch and an angioplasty guide wire (0.38 mm diameter) was introduced into the femoral artery, passed in and out three times to an approximate length of 8 mm, and left in place for 1 min to denude and dilate the artery. Subsequently, the wire was removed, and the arteriotomy site was ligated with a 9‐0 suture. The knots were then untied to restore blood flow.

To study the in vivo targeted binding of pPLV‐NO to the injured femoral arteries, DiD labeled LV‐NO or pPLV‐NO (10 mg kg^−1^ based on FTn‐Cu) were intravenously injected into rats immediately after injury. The sham rats were used as a control group. After 4 h and 24 h of administration, the fluorescence intensity at the site of injury was observed using an IVIS Spectrum imaging system (PerkinElmer, IVIS Spectrum). At 24 h, the injured femoral arteries and major organs were harvested from the rats, and ex vivo images were acquired. The mean signal intensity was quantified by measuring regions of interest (ROI).

The in vivo blood circulating profile was examined based on the previous fluorescence analysis method.^[^ ^19^ ^]^ In brief, 200 µL of DiD‐labeled LV‐NO or pPLV‐NO solution (10 mg kg^−1^ based on FTn‐Cu) were intravenously injected into healthy SD rats. Blood samples were collected at indicated time points (0, 0.5, 1, 3, 6, 12, 24, and 48 h), and observed using an IVIS Spectrum imaging system (PerkinElmer, IVIS Spectru). The mean fluorescence intensity was quantified by measuring ROI. To reduce individual differences, the experimental data at each time point were calculated relative to that of 0 h.

For the evaluation of the reparative effect, the rats were randomly assigned to four groups: sham, PBS, LV‐NO, and pPLV‐NO, receiving tail vein injections at days 0, 7, and 14 following the wire‐mediated femoral arteries injury. The treatments included PBS, LV‐NO (1 mg kg^−1^ based on FTn‐Cu), and pPLV‐NO (1 mg kg^−1^ based on FTn‐Cu). Rats in the sham group only underwent femoral arteries separation without wire‐mediated injury or any additional treatment. At day 21 post‐operation, rats were anesthetized with isoflurane and the patency and blood velocity of injured femoral arteries (n = 5 of each group) were visualized using high‐resolution ultrasound (Vevo 2100 System, Canada). Then, the rats were euthanized by injecting an overdose of pentobarbital sodium. The injured femoral arteries were photographed, fixed with 4% PFA, and embedded in optimal cutting temperature (OCT) compound (Tissue‐Tek) for frozen cross sections. The sections were stained with Verhoeff–Van Gieson (VVG) to assess neointimal hyperplasia. Immunofluorescent staining was performed with mouse anti‐CD31(Abcam, ab64543) and rabbit anti‐eNOS (Thermo Fisher Scientific, PA516887) to assess re‐endothelialization. Besides, rabbit anti‐alpha smooth muscle actin (α‐SMA, Abcam, ab124964) and mouse anti‐MYH (Abcam, ab212657) were used to assess circumferential alignment and phenotype of VSMCs. To evaluate organ toxicity of LV‐NO and pPLV‐NO, major organs (heart, liver, spleen, lung, and kidneys) were harvested at 21 days and analyzed histopathological changes by standard HE staining.

### Preparation of ePLV‐NO Modified Rat Decellularized Carotid Arteries

Rat carotid arteries were decellularized following our previous research procedures.^[^ ^27^ ^]^ Briefly, carotid arteries were harvested from SD rats and decellularized using 1% SDS and 1% Triton X‐100. RNase and DNase were employed to remove nucleic acids, and the arteries were sterilized with 0.1% peracetic acid. The ePLV‐NO was prepared using the aforementioned method and then resuspended with 2‐morpholinoethanesulphonic acid (MES) solution containing 1‐(3‐dimethylaminopropyl)‐3‐ethylcarbodiimide hydrochloride (EDC) and N‐hydroxysuccinimide (NHS). This ePLV‐NO solution was added to the intravascular cavity of the dCA, which was subsequently ligatured at both ends and incubated overnight at 4 °C. The luminal modification of the dCA with ePLV‐NO occurred via an amidation reaction between the carboxylic (‒COOH) groups on dCA and the amino (‒NH_2_) groups on ePLV‐NO. To visualize the distribution of ePLV‐NO, DiI‐labeled lipid, and Cy5‐labeled FTn were used. The modifed dCA was embedded in OCT compound (Tissue‐Tek) for frozen cross‐sections, which were then observed with confocal microscopy.

### In Vitro Hemocompatibility Evaluation of VGs

For platelet assay, rat dCAs with or without modification were longitudinally cut into two pieces. The lumen side of each sample was placed upward into a 48 well plate (n = 5) and 500 µL of rat platelet‐rich plasma (PRP), with or without NO donor (10 µm GSH and 10 µm GSNO), was added to each well. After 1 h incubation at 37 °C, the samples were rinsed three times with PBS to remove non‐adherent platelets. The samples were then cut into two equal segments along the transverse direction. One segment was fixed with 2.5% glutaraldehyde, dehydrated with a gradient of ethanol, and examined using scanning electron microscopy (SEM, Phenom Pro, Phenom‐World BV, Eindhoven, Netherlands) to observe the number as well as the morphology of adhered platelets. Another segment was fixed with 4% PFA for 20 min and stained with anti‐CD62P (Santa Cruz, US, sc‐8419) to evaluate the activation of adhered platelets. The adherent and activated platelets were observed under confocal microscopy. Five samples from each group were obtained, and five randomly selected fields from each sample were taken for statistical analysis.

For the whole blood clotting assay, fresh whole blood was obtained from a healthy rat using 5 mL vacuum blood‐collection tubes containing 3.8% (w/v) citrate. Clotting was initiated by adding 0.1 m calcium chloride (CaCl_2_) to whole blood in a ratio of 1:10. Rat dCAs with or without modification were longitudinally cut into two pieces. The lumen side of each sample was placed upward into a 48 well plate (n = 3) and incubated with 100 µL of freshly prepared recalcified whole blood with or without the addition of NO donor (10 µm GSH and 10 µm GSNO). After incubation at room temperature for 5 min, the samples were rinsed with distilled water. After removing excess water, the samples were weighed, and then the cross sections of all samples were observed under a stereomicroscope (Leica S8AP0, Germany). Quantitative results were obtained by dividing the added weight by lumen area.

### In Vivo Studies in Rat Carotid Artery Implantation Model

To evaluate the anticoagulant and re‐endothelialization ability of ePLV‐NO, male Sprague–Dawley (SD) rats (8 weeks, 250–280 g) were randomly divided into two experiment groups (n = 6 per group) to undergo implantation of either unmodified dCA or ePLV‐NO modified dCA (dCA@ePLV‐NO) (length: 1 cm, diameter: 1 mm) using a “cuff” technique as previously described.^[^ ^28^ ^]^ Briefly, rats were anesthetized with 1% pentobarbital sodium by intraperitoneal injection (30 mg kg^−1^). After exposing the left common carotid artery, it was ligated with 8‐0 silk suture two times and cut between the two ligature points. The two ends were then passed through the cuff (Portex, London, UK) and secured with artery clamps. The ends of the artery were folded from the inside out to cover the cuff body and fixed with 8‐0 silk suture. Thereafter, the two ends of dCA or dCA@ePLV‐NO (pre‐soaked in 50 U mL^−1^ heparin sodium) were anastomosed onto the ends of the carotid artery, and the vascular seams at the cuff were ligated. Blood vessel clamps were loosened, and blood flow (pulse visible) in arteries became evident. No anticoagulation drug was administered to the rats after surgery. After implantation for 2 weeks, rats were anesthetized with isoflurane and the patency of dCA or dCA@ePLV‐NO was observed by high‐resolution ultrasound (Vevo 2100 System, Canada). The dCA or dCA@ePLV‐NO was harvested and transected into two parts from the middle. One half was snap‐frozen in OCT compound (Tissue‐Tek) for frozen cross‐sectioning, while the other half was longitudinally cut into two parts. One part was prepared to observe endothelial coverage by CD31 en‐face staining according to our previous study.^[^ ^29^ ^]^ The other part was fixed in 2.5% glutaraldehyde and dehydrated with serial dilutions of ethanol to observe endothelial coverage by SEM.

### Statistical Analysis

Statistical analysis was conducted using GraphPad Prism v8.0 (GraphPad Software). Single comparisons between two independent datasets were performed using an unpaired Student's t‐test. Multiple comparisons across one variable were carried out using a one‐way analysis of variance (ANOVA), and multiple comparisons across two variables were performed using a two‐way ANOVA. All ANOVA analyses were followed by Tukey's post hoc analyses. The data are presented as means ± standard error of the mean (SEM). In all tests, a significance level of p < 0.05 was considered statistically significant.

## Conflict of Interest

The authors declare no conflict of interest.

## Supporting information

Supporting Information

## Data Availability

The data that support the findings of this study are available in the supplementary material of this article.
