# Correction to “Versatile Design of NO‐Generating Proteolipid Nanovesicles for Alleviating Vascular Injury”

**DOI:** 10.1002/advs.202501885

**Published:** 2025-02-18

Y. Yang, X. Zhang, H. Yan, R. Zhao, R. Zhang, L. Zhu, J. Zhang, A. C. Midgley, Y. Wan, S. Wang, M. Qian, Q. Zhao, D. Ai, T. Wang, D. Kong, X. Huang, K. Wang, Versatile Design of NO‐Generating Proteolipid Nanovesicles for Alleviating Vascular Injury. Adv. Sci. 2024, 11, 2401844.

https://doi.org/10.1002/advs.202401844

In Figure S9b, the figure was incorrect.


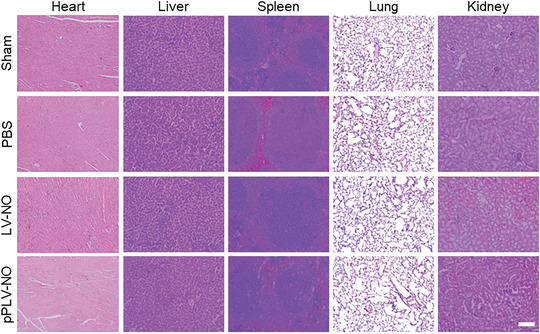


This should have read:


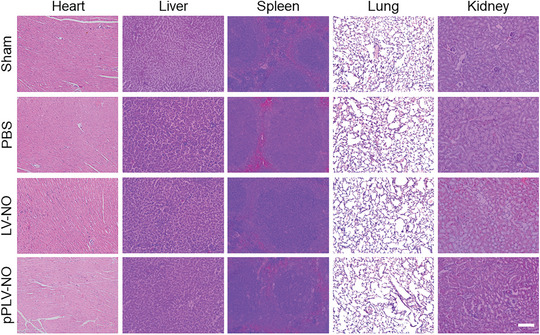


We apologize for this error.

