# Supplementary material for: Versatile Design of NO‐Generating Proteolipid Nanovesicles for Alleviating Vascular Injury
Source: Adv Sci (Weinh). 2024 Jun 17;11(31):2401844. doi: 10.1002/advs.202401844 (PMC11336937; doi:10.1002/advs.202401844)
Supplement: Supplementary file 1 — Supporting Information [file ADVS-11-2401844-s001.docx]

Supporting Information

**Versatile Design of NO-generating Proteolipid Nanovesicles for Alleviating Vascular Injury**

*Yueyue Yang, ^#^ Xiangyun Zhang, ^#^ Hongyu Yan, Rongping Zhao, Ruixin Zhang, Liuyang Zhu, Jingai Zhang, Adam C Midgley,* *Ye Wan, Songdi Wang, Meng Qian, Qiang Zhao, Ding Ai, Ting Wang, Deling Kong, ^*^ Xinglu Huang, ^*^ and Kai Wang ^*^*

Y. Yang, X. Zhang, H. Yan, R. Zhang, J. Zhang, A. C. Midgley, Y. Wan, S. Wang, M. Qian, Q. Zhao, D. Kong, X. Huang, K. Wang

Key Laboratory of Bioactive Materials for the Ministry of Education

College of Life Sciences

Nankai University

Tianjin 300071, China

E-mail: kongdeling@nankai.edu.cn (D. Kong); huangxinglu@nankai.edu.cn (X. Huang); 013053@nankai.edu.cn (K. Wang)

T. Wang

Tianjin Key Laboratory of Urban Transport Emission Research

College of Environmental Science and Engineering

Nankai University

Tianjin 300071, China

D. Ai

Department of Physiology and Pathophysiology

Tianjin Medical University

Tianjin 300070, China

L. Zhu

First Central Clinical College

Tianjin Medical University

Tianjin 300192, China

R. Zhao

School of Medicine

Nankai University

Tianjin 300071, China


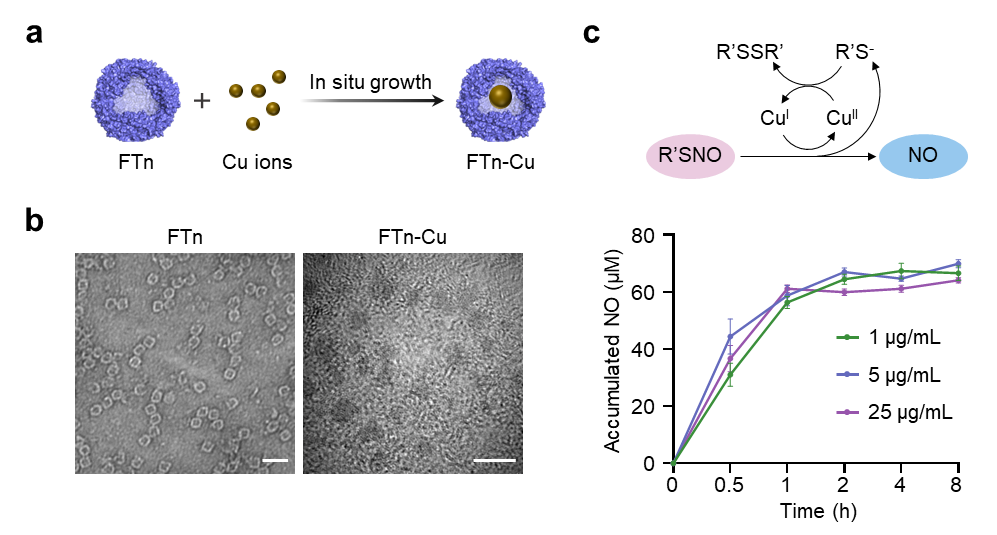


**Figure S1. Preparation and characterization of FTn-Cu nanozymes.** a) Schematic illustration of in situ integration of Cu into FTn cavity. b) TEM images of FTn protein shell with negative staining by 1% uranyl acetate (left) and FTn-Cu without negative staining (right). Scale bars are 20 nm (left) and 5 nm (right). c) Schematic illustration (top) and NO production over time in the presence of 100 μM GSNO donor catalyzed by different concentration of FTn-Cu according to the Griess assay (bottom). Data are presented as the mean ± SEM of n = 3 individual experiments.


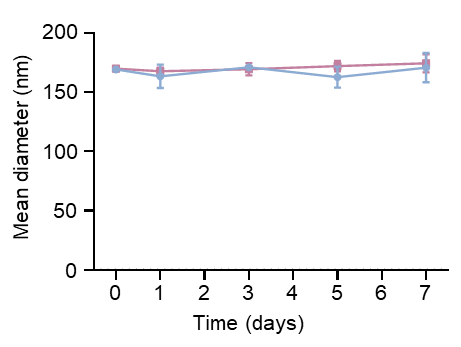


**Figure S2. Stability of NO-generating Proteolipid Nanovesicles (PLV-NO).** Size changes of PLV-NO in PBS and FBS solution at 4 ℃ over time, as determined by DLS. Data are presented as the mean ± SEM of n = 3 individual experiments.


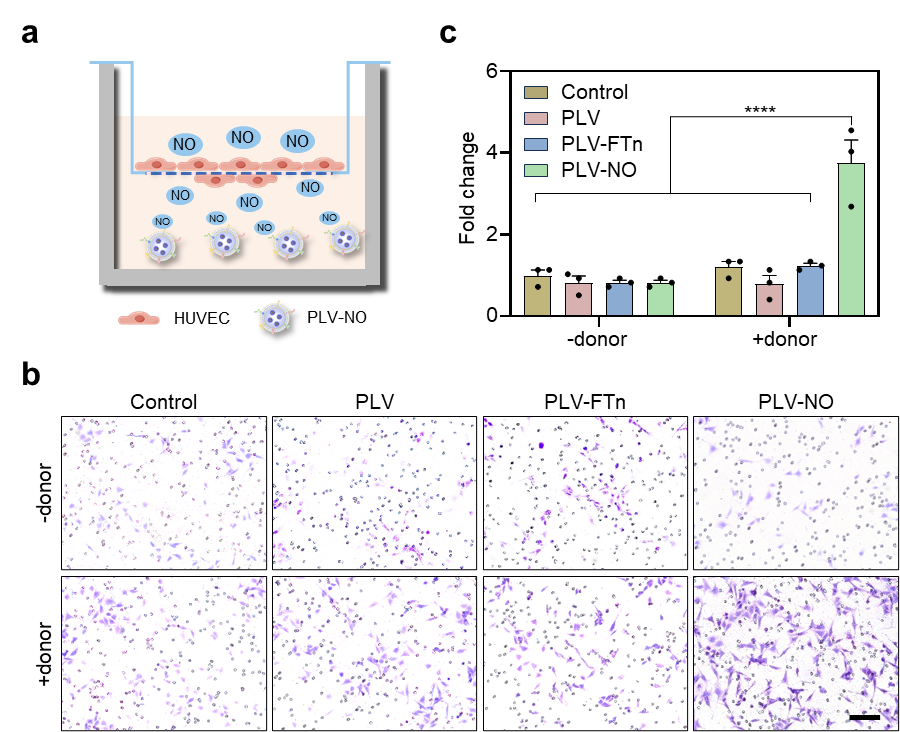


**Figure S3. Transwell assay analysis of migration capacity of HUVECs with different treatments in the absence or presence of NO donors**. a) HUVECs were plated onto upper well, medium containing different nanovesicles with or without NO donors were added into bottom well. b) Representative images and c) quantitative analysis of migrated HUVECs, three samples per each group and three randomly selected fields per sample for statistical analysis. Scale bar = 100 μm. ****P < 0.0001. All data are presented as means ± SEM. Multiple comparisons across two variables were performed using a two-way ANOVA.


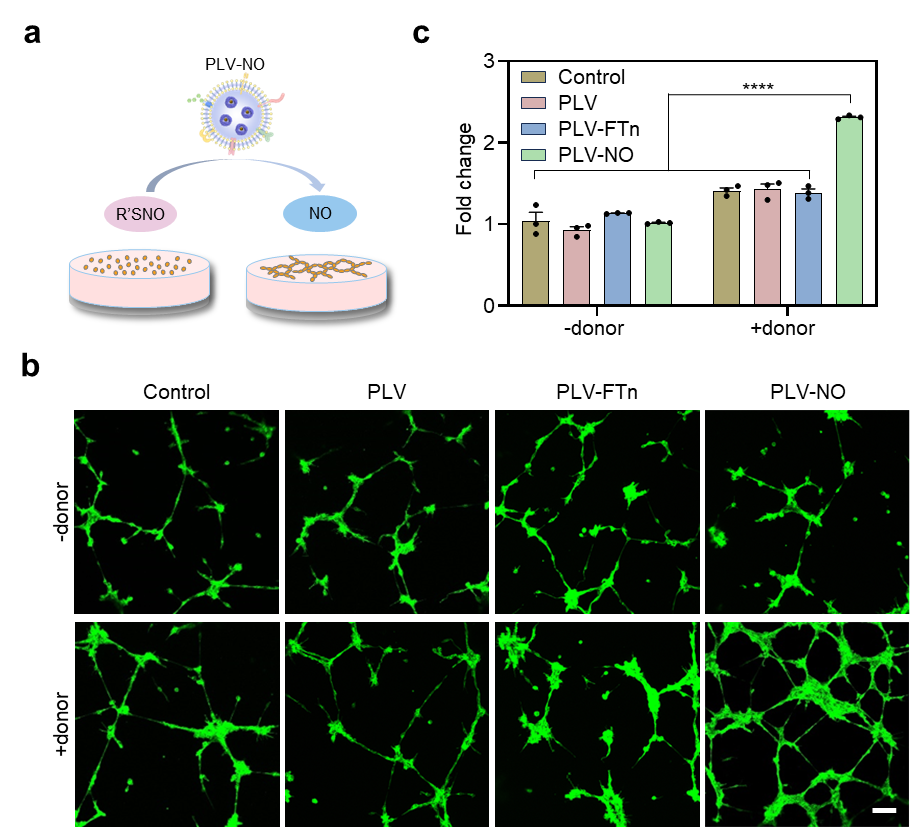


**Figure S4. Tube formation capacity of HUVECs with different treatments in the absence or presence of NO donors**. a) After plating HUVECs onto Matrigel, medium containing different nanovesicles with or without NO donors were added to incubate with HUVECs. b) F-actin staining images and c) quantification analysis of the HUVEC-lined vessels, three samples per group and three randomly selected fields per sample for statistical analysis. Scale bar = 100 μm. ****P < 0.0001. All data are presented as means ± SEM. Multiple comparisons across two variables were performed using a two-way ANOVA.


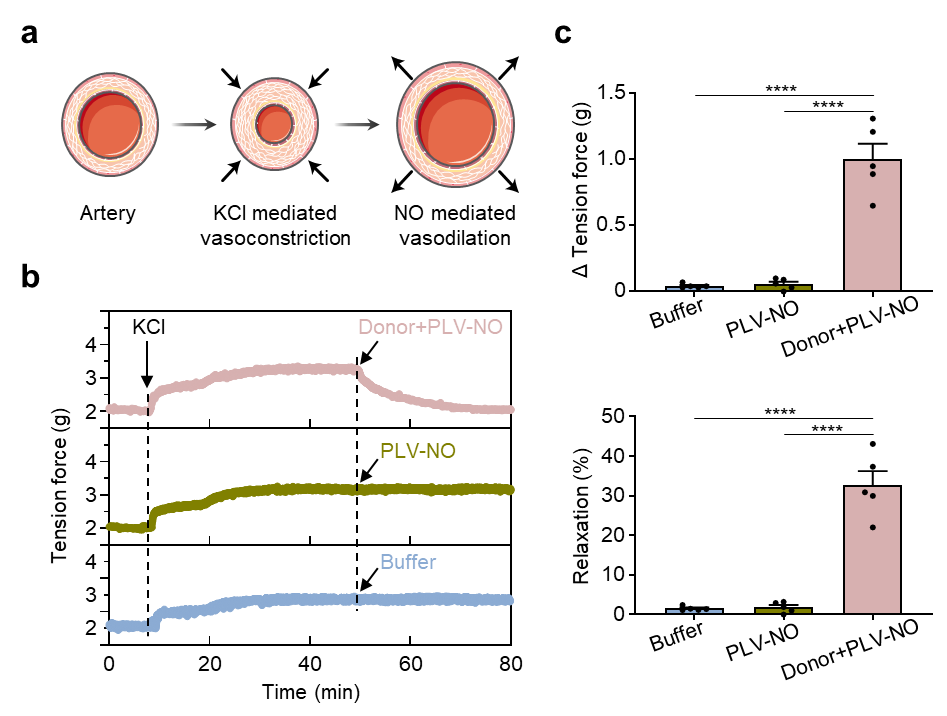


**Figure S5. Vasodilation induced by NO from PLV-NO.** a) Schematic illustration of vasodilation in response to NO from PLV-NO catalysis. Vasoconstriction was first induced by KCl, followed by addition of PLV-NO alone or accompanied by donor. b) Representative tension curves of vasodilation against time in the presence and absence of PLV-NO (equivalent to 6 μg mL^-1^ of FTn-Cu) and donor (100 μM GSNO and 100 μM GSH). c) Quantification of tension force changes and relaxation of arterial rings. All data are presented as means ± SEM, n = 5. One-way ANOVA was used to compare between three groups. ****P < 0.0001.


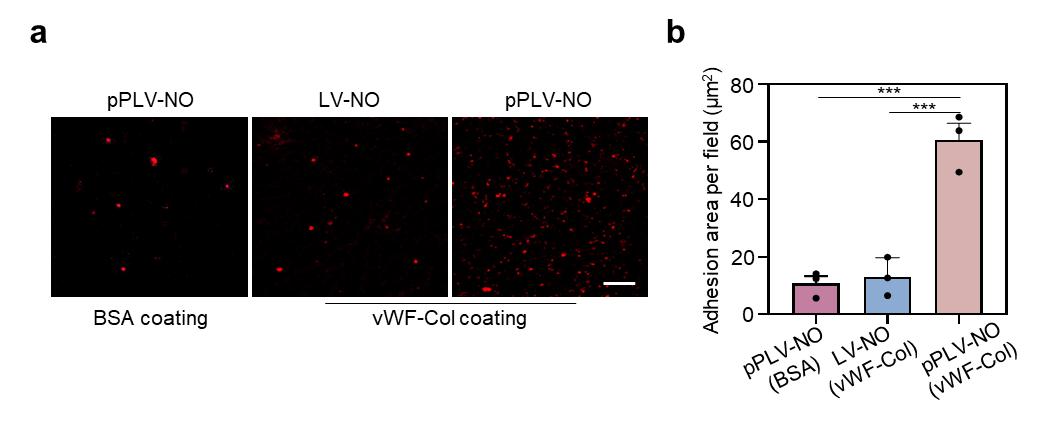


**Figure S6. Binding capacity of nanovesicles to various coatings.** a) Representative fluorescent images of DiD-labeled nanovesicles adhering on vWF-collagen or BSA coated surfaces and b) image-based quantification analysis of surface coverage by nanovesicles adhesion, three samples per group and three randomly selected fields per sample for statistical analysis. Scale bar = 10 μm. ***P < 0.001. All data are presented as means ± SEM. One-way ANOVA was used to compare between three groups.


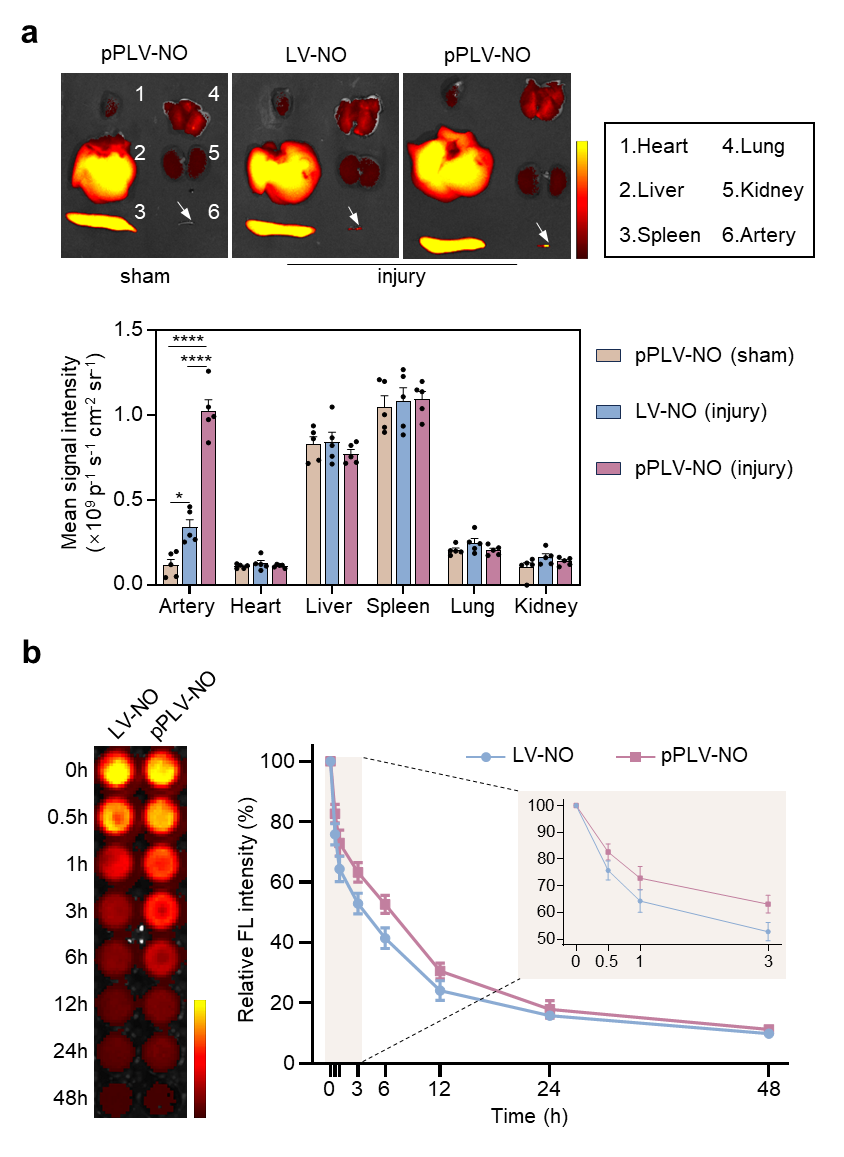


**Figure S7. Biodistribution and blood circulation of DiD-labeled LV-NO or pPLV-NO following *i.v.* administration.** a) *Ex vivo* IVIS fluorescence imaging and quantification analysis of DiD-labeled nanovesicles in different organs, five animals for each group. Arrows indicate the isolated injured vessels. *P < 0.05; ****P < 0.0001. Multiple comparisons across two variables were performed using a two-way ANOVA. b) *Ex vivo* fluorescence imaging and fluorescence intensity quantification of whole blood collected at various time points after intravenous injection of DiD-labeled LV-NO or pPLV-NO. The experimental data at each time point were calculated relative to that of 0 h, three animals for each group. All data are presented as means ± SEM.


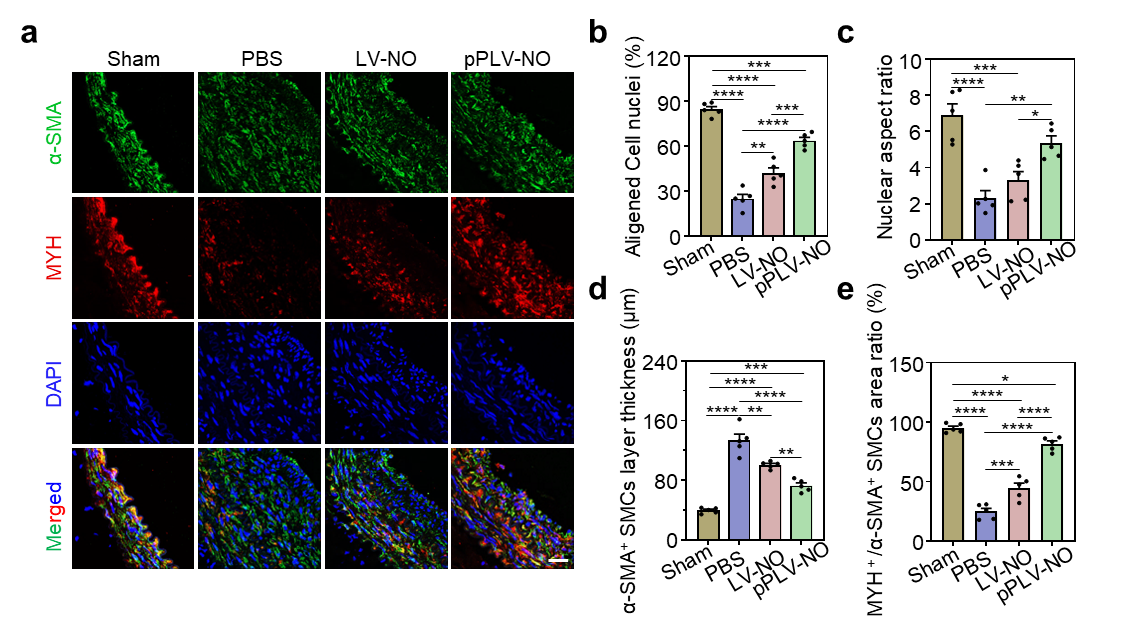


**Figure S8. Assessment of vascular smooth muscle cells through α-SMA and MYH immunostaining.** a) Immunofluorescence staining for smooth muscle cells in injured arteries using anti-α-SMA (green) and anti-MYH (red). b) Quantification of the cell nuclei alignment rate and c) nuclear aspect ratio based on the DAPI staining, indicating circumferential growth of smooth muscle cells. The nuclear aspect ratio is obtained by dividing the long axis length by the short axis length of nucleus. Five randomly selected cell nucleus per image, three high-magnification images per section, three sections per sample, and five samples per group were included for quantitative analysis. d) Quantification of thickness of the α-SMA^+^ SMCs layer to reflect SMC excessive proliferation. e) Quantitative analysis of the ratio of MYH^+^/α-SMA^+^ SMCs area, reflecting the functional condition of smooth muscle cells. Three sections per sample, five samples per group and three high-magnification images per section were included for quantitative analysis. Scale bar = 25 μm. *P < 0.05; **P < 0.01; ***P < 0.001; ****P < 0.0001. All data are presented as means ± SEM. One-way ANOVA was used to compare between four groups.


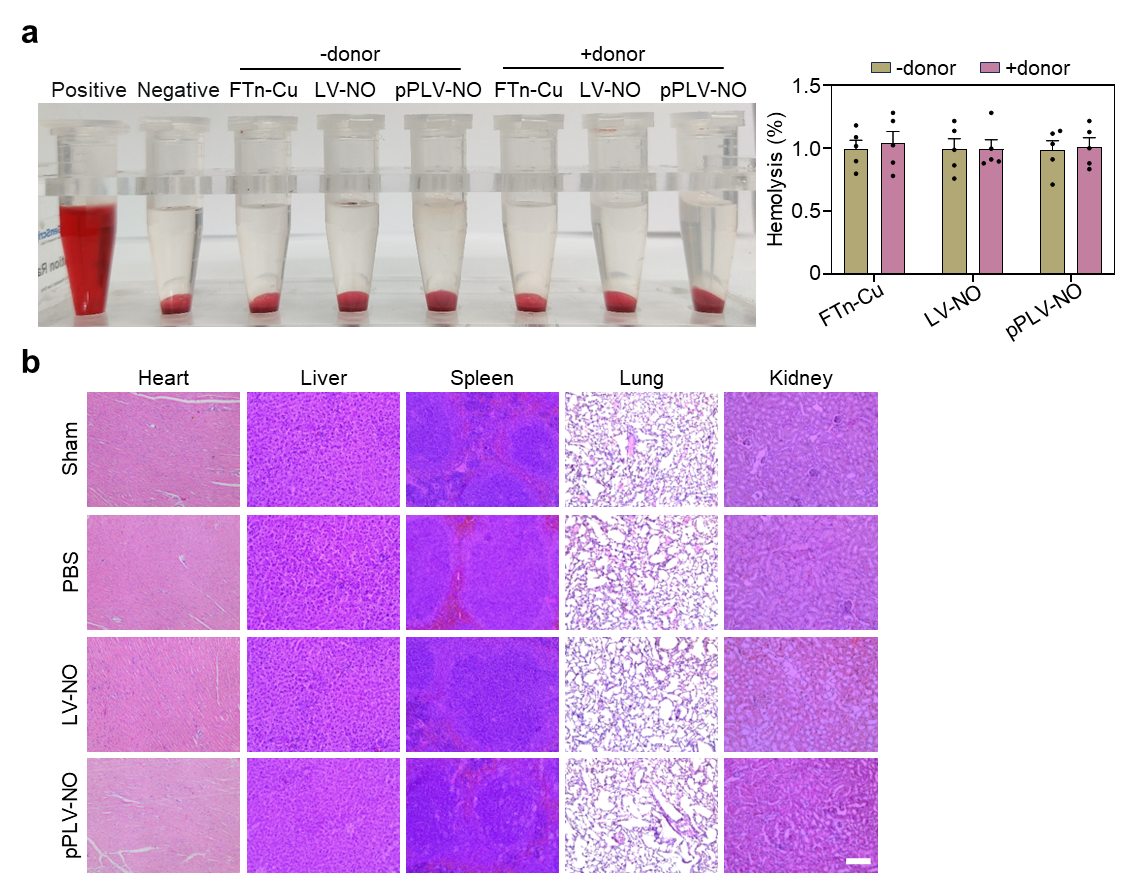


**Figure S9. Assessment of biosafety of LV-NO and pPLV-NO.** a) Brightfield photograph and quantification analysis of the hemolysis rate induced by different nanovesicles. Statistical analysis was conducted using five samples per group. Positive control: saline, Negative control: water. All data are presented as means ± SEM. Multiple comparisons across two variables were performed using a two-way ANOVA. b) Histopathological analysis of systemic toxicities in different organs isolated from the rats with or without different treatments. The slices from different organs were stained using standard H&E staining procedures. Scale bar = 200 μm.


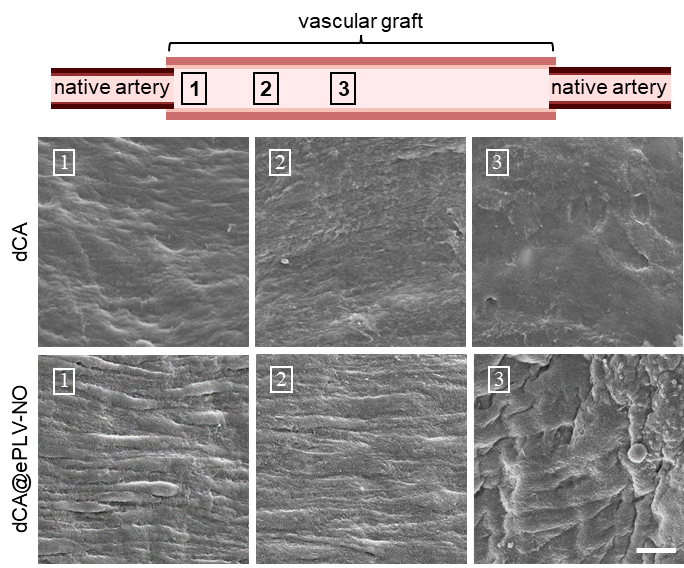


**Figure S10.** Representative surface SEM images at three different sites (anastomotic, quarter and midportion) of the explanted dCA and dCA@ePLV-NO lumen. Scale bar = 10 μm.
